# Pentapeptide Protects INS-1 Cells From hIAPP-Mediated Apoptosis by Enhancing Autophagy Through mTOR Pathway

**DOI:** 10.3389/fphar.2019.00896

**Published:** 2019-08-09

**Authors:** Jianzhen Lin, Ao Jiao, Wu Lv, Chengshuo Zhang, Yue Shi, Zhaoming Yang, Ning Sun, Xiaohang Li, Jialin Zhang

**Affiliations:** ^1^Hepatobiliary Surgery Department and Unit of Organ Transplantation, The First Hospital of China Medical University, Shenyang, China; ^2^Department of General Surgery, Liaoning Cancer Hospital and Institute, Shenyang, China

**Keywords:** human islet amyloid polypeptide, peptide inhibitor, autophagy, mTOR, islet

## Abstract

The human islet amyloid polypeptide (hIAPP), the major component of islet amyloid deposition, is one of the amyloidogenic peptides and has been associated with β cell loss and dysfunction in type 2 diabetes (T2D). Autophagy plays a central role in the clearance of hIAPP aggregates, thereby diminishing the hIAPP-induced cytotoxicity. Conversely, hIAPP has been reported to have interfering effects on the autophagy. The pentapeptide FLPNF developed in our previous study has been shown to have effects on the level of the downstream proteins of mTOR and autophagy–lysosome pathway. In the present study, the peptide FLPNF-mediated increase in autophagy flux and its underlying mechanisms, as well as its protecting effect on INS-1 cells, were investigated. Autophagy flux in INS-1 cells overexpressing hIAPP (hIAPP-INS-1 cells) markedly increased after exposure to peptide FLPNF for 24 h and peaked at a concentration of 200 µM. Peptide FLPNF enhanced the autophagy by inhibiting the mTORC1 activity. Flow cytometry results showed the peptide FLPNF bind to mammalian target of rapamycin (mTOR), and further molecular docking analysis revealed a direct interaction between peptide FLPNF and the FRB domain of mTOR. Meanwhile, both peptide FLPNF and rapamycin significantly decreased the hIAPP-induced apoptosis, whereas 3-MA increased the apoptosis. Furthermore, peptide FLPNF reduced the hIAPP oligomer and improved the hIAPP-INS-1 cells insulin release function at high glucose concentration. Taken together, the peptide FLPNF decreased the hIAPP oligomer *via* upregulating autophagy by inhibiting mTORC1 activity, thus protecting the INS-1 cells from hIAPP-induced apoptosis and improving the insulin release function of INS-1 cells.

## Introduction

Islet amyloid polypeptide (IAPP), also known as amylin, is a 37 amino acids peptide hormone (Raleigh et al., 2017) which is co-secreted and co-packaged with insulin by β cells in response to nutrients (Hay et al., 2015). Hitherto, IAPP has been found in most of the mammals with conserved amino acid sequence across evolution. However, six differences were detected in the amino acid sequence between human IAPP (hIAPP) and rodent IAPP (rIAPP), which makes hIAPP highly susceptible to misfolding and self-aggregation, thereby leading to oligomer formation and amyloid deposition (Westermark et al., 2011). Due to this difference in amino acid sequence of IAPP, islet amyloid deposition is detected in approximately 90% of patients with type 2 diabetes but not in rodent diabetes (Kahn et al., 1999). Monomeric hIAPP is non-cytotoxic, while amyloid deposition and oligomers formed during aggregation can lead to impaired function of islet β cells and increased apoptosis of β cells, which aggravates diabetes (Wijesekara et al., 2015; Raleigh et al., 2017). Therefore, strategies that prevent the accumulation of hIAPP oligomer or promote the degradation of oligomer represent a novel therapy for T2D (Costes, 2018).

Autophagy, the way by which cells degrade damaged organelles and misfolded proteins, is one of intracellular quality control system (Williams et al., 2006; Mizushima et al., 2008; Mizushima and Komatsu, 2011). Current studies have indicated that transgenic mice expressing hIAPP in β cells with autophagy deficiency resulted in the arise of diabetes and hIAPP aggregates accumulation in pancreatic islet; these phenomena were not observed in mice merely expressing hIAPP (Kim et al., 2014; Rivera et al., 2014). While autophagy deficiency obstructed the hIAPP clearance and aggravated hIAPP-induced cytotoxicity, hIAPP oligomer disturbed the autophagy pathway in β cells (Kiriyama and Nochi, 2018). The elevated level of LC3-II represented an increased autophagosomal number as detected in islet of hIAPP transgenic mice (Rivera et al., 2011; Kim et al., 2014). However, this increased in autophagosomal number might be attributed to the autophagy blockage rather than the enhanced autophagy flux because the level of p62 was also increased in the islet of such transgenic mice (Rivera et al., 2011; Kim et al., 2014; Rivera et al., 2014). Thus, it can be speculated that hIAPP oligomer impaired the autophagy, resulting in profound accumulation of hIAPP aggregates. This feed-forward loop might lead to further β cells damage and loss, thus aggravate diabetes. Moreover, autophagy enhancer rapamycin has been reported to improve β cells function, accompanied by increased islet autophagy *via* inhibiting mTOR activity, reduce hIAPP oligomer accumulation, and decrease cellular apoptosis (Rivera et al., 2014). Consequently, studies on small molecules that inhibit mTOR activity to promote autophagy have emerged (Veverka et al., 2008; Morad et al., 2013; Bajer et al., 2014; Kim and Guan, 2015; Fan et al., 2017).

Another strategy to reduce hIAPP aggregates is to prevent hIAPP misfolding by hIAPP inhibitors, which primarily include small molecular compounds (Pithadia et al., 2016; Franko et al., 2018), metal ions (Su et al., 2017), and short peptide inhibitors. Compared to the other types of inhibitors, short peptide inhibitors present the advantages of lower cytotoxicity, cost-efficiency, easy synthesis, and the ability to recognize and bind to hIAPP misfolded region. Reportedly, the peptides inhibitors, such as SNNFGA, GAILSS (Scrocchi et al., 2002), NYGAILSS, and NFGAILFF (Porat et al., 2004), inhibited the hIAPP aggregation *in vitro*, while D-ANFLVH (Wijesekara et al., 2015) reduced the hIAPP deposits *in vivo*. Although the above *in vivo* and *in vitro* studies confirmed that short peptide inhibitors could effectively alleviate the hIAPP aggregation, the underlying mechanisms are yet to be elucidated, especially the effect of short peptides on autophagy flux.

In our previous study, a pentapeptide FLPNF (Phe-Leu-Pro-Asn-Phe) that contains five amino acids has been designed and was shown to inhibit hIAPP aggregation *in vitro* (Shi et al., 2019). Furthermore, Label-free quantitative proteomics analysis showed that after exposure to 200 µM peptide FLPNF for 24 h, IGF2 (Insulin-like Growth Factor 2) was down-regulated and NPTX1 (Neuronal Pentraxin-1) was up-regulated, both of which are downstream proteins of mTOR. And the DVL2 (Dishevelled 2), which can be degraded by the autophagy-lysosome pathway, was also down-regulated. The changes of the level of above proteins were similar to the effect of inhibition of mTOR pathway by rapamycin (Gao et al., 2010; Tyburczy et al., 2010; Dai et al., 2011). However, the mechanism of how peptide FLPNF affect autophagy pathway remains unclear. Therefore, the aim of the present study is to investigate the peptide FLPNF-mediated increases in the autophagy flux and its underlying mechanisms, as well as its protecting effect on hIAPP-induced cytotoxicity.

## Materials and Methods

### Compounds and Reagents

**Figure 1:**
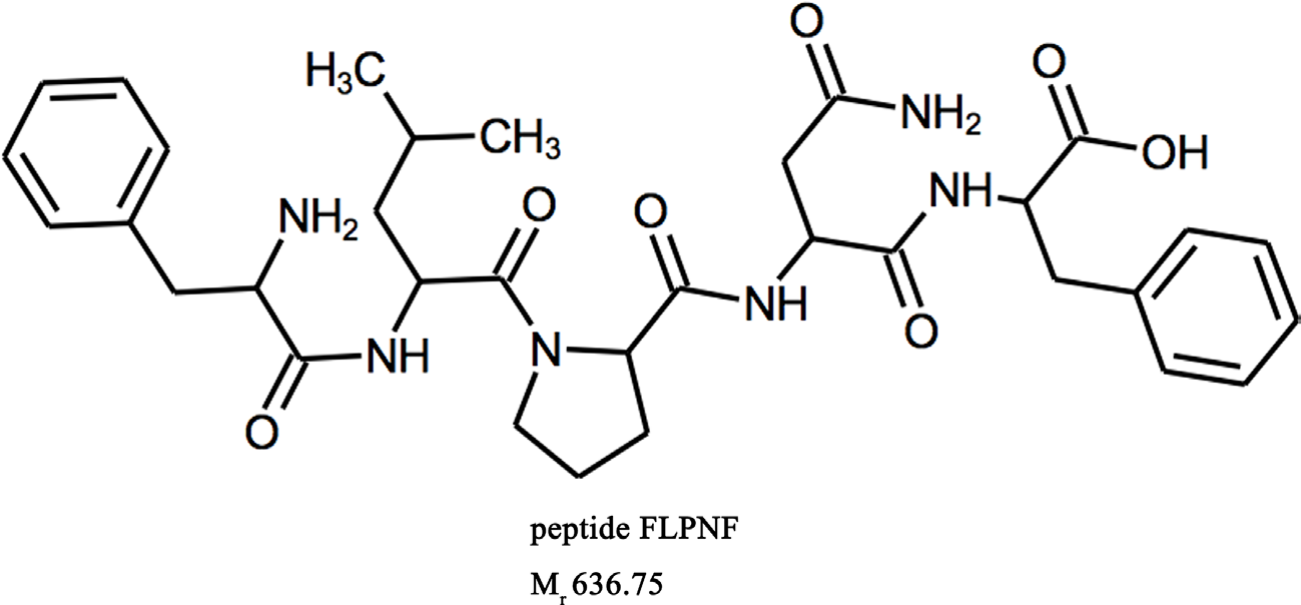
Molecular structure of peptide FLPNF.

Peptide FLPNF (**Figure 1**), D-ANFLVH and NFGAIL, were synthesized by ChinaPeptides Co., Ltd (Shanghai, China, purity >98%) as previously described (Shi et al., 2019). Cell-penetrating peptide HIV-TAT(48–57) (GRKKRRQRRR) was fused to peptide FLPNF at the N terminal to enhance cellular permeability, and the TAT linked FLPNF was more effective in inducing autophagy (**Supplementary Figure 1**). The linker GSG (Gly–Ser–Gly) was added between TAT peptide and peptide FLPNF (GRKKRRQRRR-GSG-FLPNF) to prevent potential interaction between the two peptides and enhance peptide flexibility (Sutton et al., 2019). The synthetic peptides were solubilized in dimethyl sulfoxide (DMSO) at 100 mM according to the manufacturer’s instructions. Rapamycin, 3-Methyladenine (3-MA), and bafilomycin-A1 (Baf-A1) were obtained from Solarbio (Beijing, China). Insulin-like growth factor-1 (IGF-1) and protein phosphatase 2A (PP2A) inhibitor okadaic acid (OA) were purchased from sigma (85580C and O8010, respectively). Anti-β-actin, anti-Bax, anti-phospho-AKT, and anti-AKT antibodies were purchased from Proteintech, while anti-Bcl-2 antibody was from Santa Cruz Biotechnology. All other primary antibodies were obtained from Cell Signaling Technology (CST) if not indicated otherwise.

### Cell Culture

INS-1 cells were obtained from Bioleaf Biotech (Shanghai, China). Cells were cultured in RPMI 1640 (Gibco, CA, USA) containing 11.1 mM glucose, 10% (v/v) fetal bovine serum (FBS) (10099141, Gibco, Australia), 50 µM β-mercaptoethanol (M3148, Sigma), 10 mM HEPES (H1090, Solarbio), 2 mM L-glutamine (G0200, Solarbio), 1 mM sodium pyruvate (SP0100, Solarbio), 100 U/ml penicillin (P1400, Solarbio), and 100 µg/ml streptomycin(P1400, Solarbio), as described previously (Jiao et al., 2018). Human embryonic kidney 293 (HEK-293) cells were obtained from the China Center for Type Culture Collection (CCTCC, Wuhan, China) and cultured in Dulbecco’s modified essential media (10564029, DMEM) (Gibco) supplemented with 10% (v/v) FBS (10099141, Gibco), 100 U/ml penicillin and 100 µg/ml streptomycin (P1400, Solarbio) (Zhang et al., 2018). The cells were maintained at 37°C in a humidified atmosphere containing 95% air and 5% CO_2_.

### Cell Transduction

Recombinant human IAPP and rat IAPP adenovirus (hIAPP and rIAPP, respectively) were generated by GeneChem (Shanghai, China), as described previously (Shigihara et al., 2014). For cells transduction, INS-1 cells were plated at a density of 5 × 10^5^ cells/well in 6-well plates (354595, Corning, NY, USA), and cultured for 24 h. Subsequently, the cells were transduced with hIAPP or rIAPP adenovirus at 20 MOI for 6 h according to the manufacturer’s instructions (hereafter referred to as hIAPP-INS-1 cells and rIAPP-INS-1 cells, respectively). Tandem fluorescent-tagged LC3 (mRFP-GFP-LC3) adenovirus was obtained from HanBio Technology Co., Ltd. (Shanghai, China). Lentivirus expressing myristylated, i.e., constitutively active, Akt (myr-Akt) was generated by OBiO Technology Co., Ltd. (Shanghai, China). The transduction was performed according to the manufacturer’s instructions, followed by further experiments after 48 h.

### Western Blot

Total cell extract were analyzed by Western blot as described previously (Jiao et al., 2018). Briefly, the total protein from treated cells was extracted using radio-immunoprecipitation assay (RIPA) lysis buffer (P0013B, Beyotime, Shanghai, China) supplemented with protease inhibitors (P1006, Beyotime) and phosphatase inhibitors (P1046, Beyotime). The protein concentrations were measured using a Bicinchoninic Acid (BCA) protein assay kit (P0010S, Beyotime). An equivalent of 20 micrograms of total protein extract were resolved by 10% or 12% sodium dodecyl sulfate-polyacrylamide gel electrophoresis (SDS-PAGE) (KGP113K, KeyGen Biotech. Co. Ltd., Nanjing, China), then the gels were transferred to PVDF membranes (Millipore, Temecula, CA, USA). Subsequently, the polyvinylidene difluoride (PVDF) membranes were blocked with 5% nonfat dried milk solubilized in TBST for 2 h and probed with primary antibodies against LC3, p62, p-S6 (Ser240/244), S6, p-P70S6K (Thr389), P70S6K, p-GSK3β, GSK3β, p-AKT (Ser473), AKT, cleaved caspase-3, Bax, Bcl-2, and β-actin overnight at 4°C. Then, the membranes were incubated with secondary antibodies for 2 h at room temperature. The immunoreactive bands were revealed using ECL Western blot protocol (P0018, Beyotime). The intensity of bands was measured using the Image Lab 5.0 software.

### Confocal Scanning Laser Microscopy

After transduction, hIAPP-INS-1 cells expressing mRFP-GFP-LC3 were plated at a density of 2 × 10^5^ cells/well in 28.2 mm culture dish with glass bottom (801002, NEST, Wuxi, China). After attachment, the transduced cells were treated with DMSO, 3-MA, rapamycin, and peptide FLPNF, respectively for additional 24 h. The change in autophagy flux was assessed as the number of GFP, RFP, and merged yellow points (point/cell) using a confocal scanning laser microscope (NIKON, Shanghai, China), as described previously (Li et al., 2017).

### Transmission Electron Microscopy

hIAPP-INS-1 cells were seeded in 6 cm cell culture dish (356401, Corning) and culture for 24 h before treatment. After treatment with DMSO, 3-MA, rapamycin, and peptide FLPNF for 24 h, respectively, the cell pellets were immediately fixed in 2.5% glutaraldehyde at 4°C for 4 h, followed by post-fixation with 1% osmium tetroxide for 2 h. Then the cells were dehydrated with acetone and embedded in Spurr’s epoxy resin. Thin sections (60–70 nm thickness) were obtained with ultramicrotome (UC7, Leica, Germany), stained with lead citrate, and examined under a HT7700 transmission electron microscope (TEM) (Hitachi, Tokyo, Japan). Autophagosomes are represented by double membrane vesicles. Quantification was performed manually by counting the average number of autophagosomes in 30 randomly selected cells for each group as described previously (Sutton et al., 2019).

### Flow Cytometry Analysis the Binding of Peptide With mTOR

In order to investigate the binding of peptide FLPNF with mTOR, the plasmids that express Flag-labeled mTOR [pcDNA3-Flag mTOR wt was a gift from Jie Chen (Addgene plasmid # 26603; http://n2t.net/addgene:26603; RRID: Addgene_26603)] were transfected into HEK-293 cells. The lysates extracted from transfected HEK-293 were then incubated with the anti-Flag magnetic beads (B26102, Bimake, Houston, USA) on roller at room temperature for 1 h, followed by three-times wash with TBST. 200 μM FITC-labeled peptides were incubated with these Flag-mTOR coated beads (1 × 10^5^) on a roller at room temperature for 1 h and then washed three times with TBST. Finally, the fluorescence intensity of the magnetic beads was analyzed by flow cytometry (FACSCalibur; Becton Dickinson).

### Molecular Modeling and Docking Analyses

The molecular docking analyses was conducted to investigate the binding mode between peptide FLPNF and FRB domain of mTOR using Autodock Vina 1.1.2 (Trott and Olson, 2010) as previously described (Shi et al., 2019). Briefly, the three-dimensional (3D) structure of FRB domain was extracted from 3D structure of the FKBP12-rapamycin-FRB ternary complex downloaded from the RCSB Protein Data Bank (PDB ID: 1NSG). The 3D structure of peptide FLPNF was built by the PyMOL 1.7.2.1 package. The docking input files were generated by the AutoDockTools 1.5.6 package. Peptide FLPNF was prepared for docking by merging nonpolar hydrogen atoms and defining rotatable bonds. The search grid of the FRB domain site was identified as center x = -9.572, center y = 26.922, and center z = 36.187 with dimension size x = 20, size y = 20, and size z = 20. In order to increase the docking accuracy, the value of exhaustiveness was set to 20. For Vina docking, the default parameters were used if it was not mentioned. The best scoring pose as judged by the Vina docking score was chosen and visually analyzed using the PyMOL 1.7.2.1 software.

### Immunofluorescence Assay

The successive cell slices were fixed in 2% paraformaldehyde (w/v) (Servicebio) for 10 min, rinsed in TBSTx buffer (Beyotime), and blocked for 1 h with TBSTx buffer supplemented with 5% bovine serum albumin (BSA) (w/v) (Solarbio). Subsequently, the sections were incubated with FITC-conjugated oligomer-specific (A11) antibody (1:100; ab183460, Abcam) overnight at 4°C (Shigihara et al., 2014). Subsequently, the sections were incubated with DAPI (Beyotime) for 5 min before imaging using an inverted fluorescence microscope. The toxic hIAPP oligomers were indicated as perinuclear or vesicle-like punctas in cytoplasm within β cells (Lin et al., 2007). For determining the β cells positive for A11 (cells with bright green A11 signal/counted cells × 100%), 30 cells/sample were enumerated manually.

### Assessment of Cell Viability

Cell viability was assessed using CCK-8 kit (C0037, Beyotime). INS-1 cells were seeded at a density of 1 × 10^4^/well in 96-well plates (100 µl/well). After treatment, 10 µl CCK-8 was added to each well and cells were incubated for an additional 1 h. Then, the absorbance at 450 nm was detected using a microplate reader. The viability ratio was calculated according to the following formula: Viability ratio = (absorbance of experimental group − absorbance of blank group/absorbance of control group − absorbance of blank group) × 100%.

### Assessment of Insulin Release and Total Insulin Content

Cells after treatment were preincubated for 60 min at 37°C in Krebs–Ringer bicarbonate HEPES buffer (KRBH) (115 mM NaCl, 4.7 mM KCl, 1.28 mM CaCl_2_, 1.2 mM MgSO_4_, 10 mM NaHCO_3_, and 20 mM HEPES) containing 1.1 mM glucose supplemented with 1 mg/ml BSA. Then, the cells were exposed to 1.1 or 20.0 mM glucose with or without DMSO, 3-MA, rapamycin, or peptide FLPNF for 60 min. Subsequently, the supernatants were collected from each well, and secreted insulin was determined using the Ultrasensitive Rat Insulin ELISA kit (10-1251-01, Mercodia, Sweden) according to the manufacturer’s instructions. Insulin secretion data were normalized to total insulin content of the cells collected from each well using RIPA buffer (Beyotime). Also, total insulin content was determined by ELISA (Mercodia) (Jiao et al., 2018).

### Statistical Analysis

Graph-Pad Prism 6 software (GraphPad Software, Inc., USA) was used for all the statistical analyses. Data are presented as the mean ± standard deviations (SD). The significant differences between data sets were analyzed by Student’s unpaired t-test with two-tailed *p*-values and one-way ANOVA, followed by Tukey’s multiple comparisons test. A *p*-value < 0.05 was considered to be statistically significant. All experiments were performed at least three times.

## Results

### Peptide FLPNF Increases the Number of hIAPP-INS-1 Intracellular Autophagosomes

**Figure 2:**
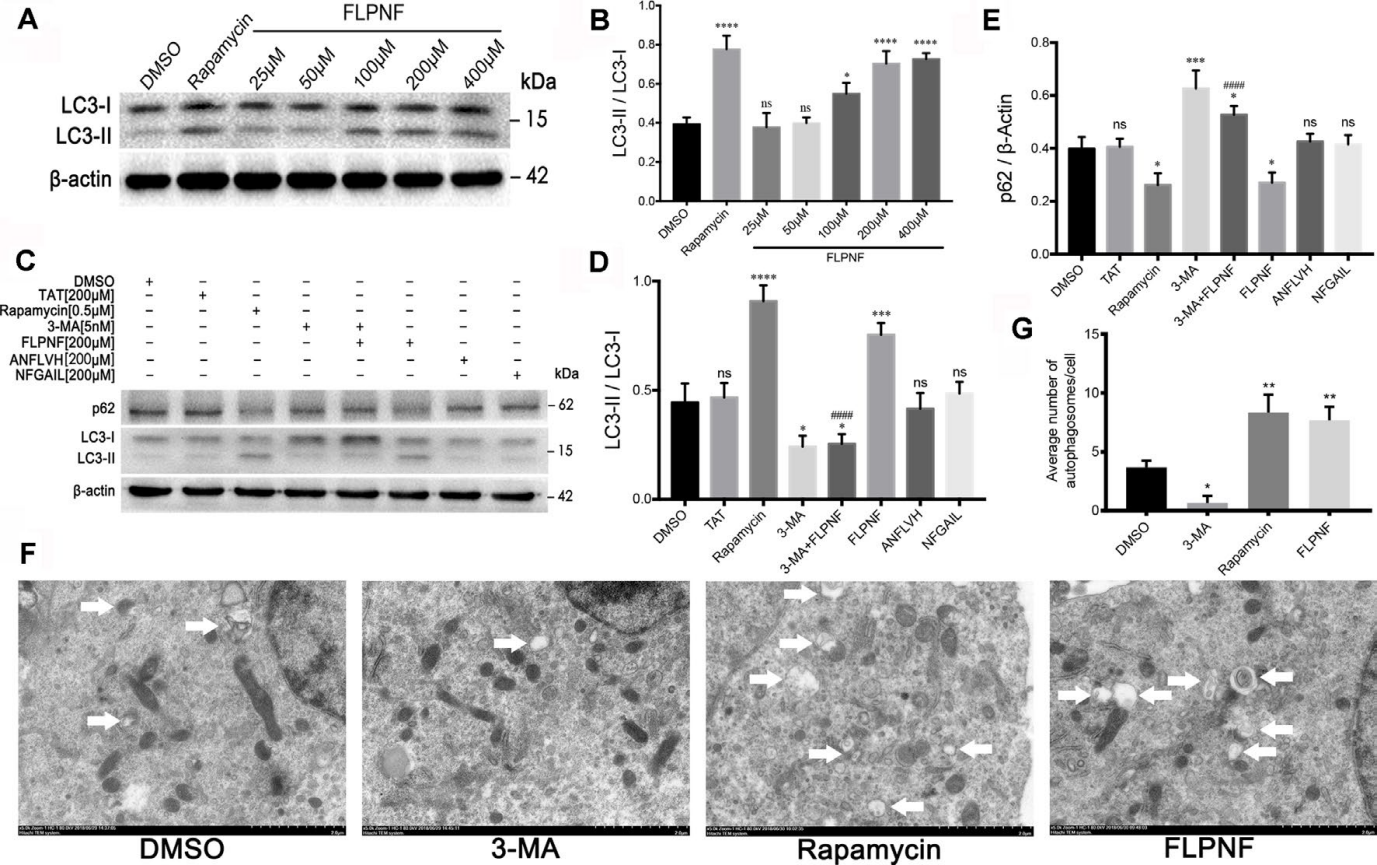
Peptide FLPNF significantly increases the autophagosomal number in hIAPP-INS-1 cells. **(A)** Western blot analysis revealed changes in LC3-I and LC3-II at different concentration of the peptide FLPNF. β-actin was used as loading control. **(B)** The graph represents the quantification of LC3-II protein levels (n = 4). *p < 0.05, ****p < 0.0001, and ns *vs*. DMSO group. **(C)** Protein levels of LC3 and p62 were assessed by Western blot in hIAPP-INS-1 cells after exposure to 200 µM peptide FLPNF for 24 h. Rapamycin (0.5 µM for 6 h), 3-MA (5 nM for 6 h), peptide ANFLVH (200 µM for 24 h), and peptide NFGAIL (200 µM for 24 h) were used as a control. **(D)** and **(E)** The graph represents the quantification of LC3-II and p62 protein levels, respectively (n = 4). *p < 0.05, ***p < 0.001, ****p < 0.0001, and ns *vs*. DMSO group. ^####^ *p* < 0.0001 *vs*. peptide FLPNF group. **(F)** Autophagy was evaluated using TEM, autophagosome are represented by double membrane vesicles and denoted with white arrow. Magnification 50,000×. **(G)** Quantification of the average number of autophagosomes per cell was determined by counting manually in 30 randomly selected cells for each group (n = 3). *p < 0.05 and **p < 0.01 *vs*. DMSO group.

In order to investigate whether peptide FLPNF affects the cellular autophagy flux, hIAPP-INS-1 cells were exposed to 0–400 µM peptide FLPNF for 24 h. The hIAPP fibril formation enhancer peptide NFGAIL (Scrocchi et al., 2002), peptide D-ANFLVH (Wijesekara et al., 2015), and rapamycin were used as control. LC3-II, which is the most widely detected protein for monitoring autophagy, was determined by immunoblot analysis to reveal the intracellular autophagosomal number (Klionsky et al., 2016). We found that the expression of LC3-II increased with the increasing concentration of peptide FLPNF, and peaked at a concentration of 200 µM (**Figures 2A, B**). Therefore, we used the concentration of 200 µM for subsequent experiments. Furthermore, the peptide FLPNF-mediated increase in the expression of LC3-II and decrease in the expression of p62 were repressed by autophagy-promotion inhibitor 3-MA (**Figures 2C–E**), which indicated that peptide FNPNF increased the intracellular autophagosomal number. However, neither the peptides NFGAIL nor D-ANFLVH affected the autophagosomal number (**Figures 2C–E**). TEM (Transmission electron microscopy) is another well-characterized method to image autophagic vacuoles. Thus, TEM was conducted to verify these results and shown that the number of autophagosome was significantly increased after exposure to 200 µM peptide FLPNF for 24 h and to 0.5 µM rapamycin for 6 h (**Figures 2F, G**). These data suggested that peptide FLPNF distinctly increased the autophagosomal number in hIAPP-INS-1 cells. In order to exclude the unexpected cytotoxicity of peptide FLPNF, INS-1 cells were exposed to different concentration of peptide FLPNF for different time. Peptide FLPNF did not cause a change in viability of INS-1 cells even after exposure to 400 µM for 96 h (**Supplementary Figure 2**).

### Peptide FLPNF Induces hIAPP-INS-1 Cells’ Autophagy

**Figure 3:**
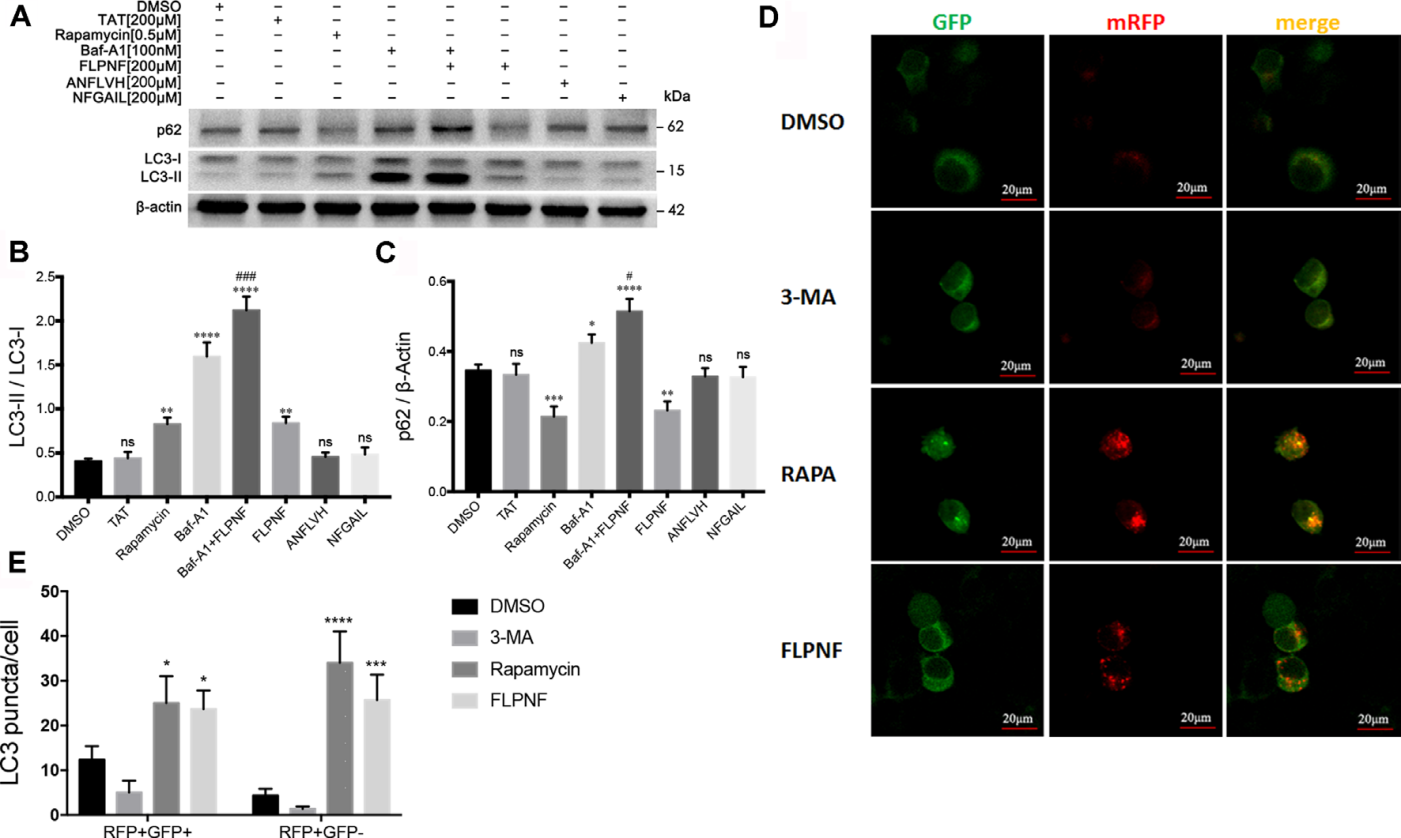
Peptide FLPNF significantly induces the autophagy flux in hIAPP-INS-1 cells. **(A)** Protein levels of LC3 and p62 were assessed by Western blot in hIAPP-INS-1 cells after exposure to 200 µM peptide FLPNF for 24 hours. Autophagosome-lysosome fusion inhibitor bafilomycin-A1 (100 nM) was used to confirm the peptide FLPNF-induced autophagy flux. **(B)** and **(C)** The graph represents the quantification of LC3-II and p62 protein levels, respectively (n = 4). *p < 0.05, **p < 0.01, ***p < 0.001, ****p < 0.0001, and ns *vs*. DMSO group. ^#^ *p* < 0.05 and ^###^ *p* < 0.001 *vs*. Baf-A1 group. **(D)** Representative confocal fluorescent images of hIAPP-INS-1 cells-expressing mRFP-GFP-LC3. Yellow puncta represent autophagosomes and red puncta for autolysosomes, which were quantified in the **(E)** (n = 4). *p < 0.05, ***p < 0.001, and ****p < 0.0001 *vs*. DMSO group.

Autophagy flux involves the formation of autophagosomes, fusion with lysosome to form autolysosomes, and cargos degradation. The increased in the autophagosomal number might result from either autophagy induction or autophagy blockage. To clarify whether autophagy flux enhanced, we verified the peptide FLPNF-induced LC3-II increase under the pre-incubation of an autophagosome-lysosome fusion inhibitor, bafilomycin-A1. As expected, LC3-II was markedly increased after bafilomycin-A1 treatment because bafilomycin-A1 blocked LC3-II degradation. Peptide FLPNF-induced autophagy flux was validated since the peptide FLPNF combined with bafilomycin-A1 treatment further increased the level of LC3-II as compared to bafilomycin-A1 or peptide FLPNF treatment alone, and p62 which is the selective substrate of autophagy was degraded after peptide FLPNF treatment (**Figures 3A–C**). Moreover, the autophagy flux monitored by mRFP-GFP-LC3 demonstrated that the number of autophagosomes (RFP+GFP+ dots) and autolysosomes (RFP+GFP- dots) increased significantly after exposure to 200 µM peptide FLPNF for 24 h (**Figures 3D, E**), revealing the enhancement of autophagy flux.

### Peptide FLPNF Interferes With Akt-mTOR Cascade

**Figure 4:**
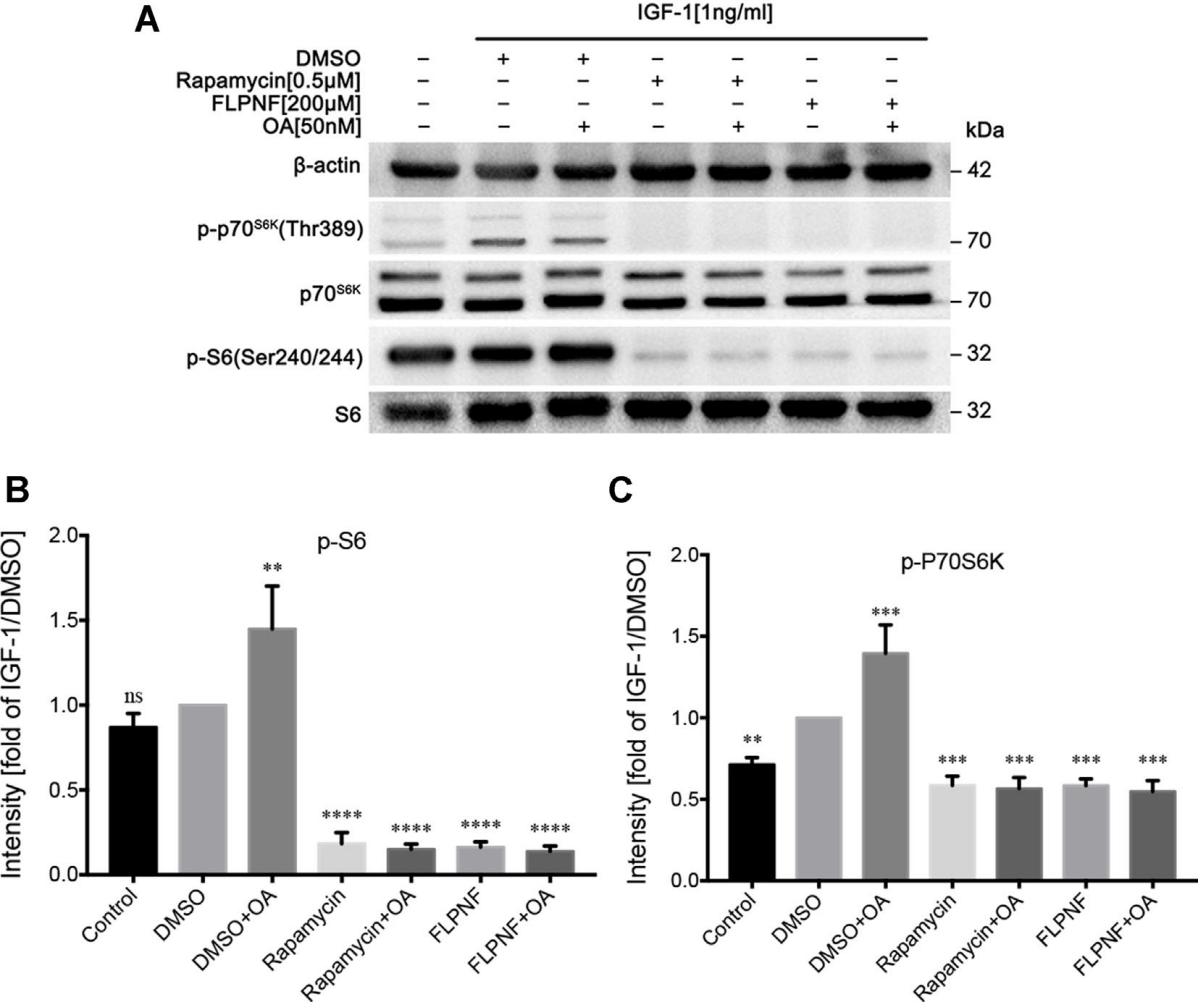
Peptide FLPNF inhibits IGF-1-induced activation of Akt-mTOR signaling. **(A)** Protein levels of phosphorylated S6 and P70S6K were assessed by Western blot in hIAPP-INS-1 cells after exposure to 200 µM peptide FLPNF for 24 h. The protein phosphatase 2A inhibitor okadaic acid (50 nM) was added for the final 60 min of the incubation (lanes 3, 5, and 7) before IGF-1 (1 ng/mL) was added for 20 min. **(B)** and **(C)** The graph represents the quantification of phosphorylated S6 and P70S6K protein levels, respectively (n = 4). The band intensities were quantified and statistically analyzed relative to IGF-1/DMSO-treated cell. **p < 0.01, ***p < 0.001, and ****p < 0.0001 *vs*. IGF-1/DMSO-treated group.

mTOR, downstream in the PI3K signaling cascade, is a major regulator of cellular autophagy flux. To clarify the specific mechanism underlying the peptide FLPNF-induced autophagy, we performed Western blot to find out the molecular target of peptide FLPNF within the PI3K-Akt-mTOR-P70S6K pathway. After serum starvation for 24 h, the hIAPP-INS-1 cells were exposed to peptide FLPNF or rapamycin followed by stimulation with 1ng/mL IGF-1 for 20 min to activate the PI3K signaling. The pre-treatment hIAPP-INS-1 cells with 200 µM peptide FLPNF for 24 h or with 0.5 µM rapamycin for 6 h efficiently blocked the IGF-1-induced phosphorylation of S6 (**Figures 4A, B**). In addition, both reagents inhibited the phosphorylation of the upstream P70S6K (**Figures 4A, C**), indicating the peptide FLPNF, similar to rapamycin, acted upstream of P70S6K. However, the decreased level of phosphorylated P70S6K resulted from either inhibited its phosphorylation or promoting the dephosphorylation by PP2A. Therefore, we treated hIAPP-INS-1 cells with rapamycin or peptide FLPNF, and added 50 nM of PP2A inhibitor, OA, for the final hour before stimulation with IGF-1. Neither rapamycin-mediated nor peptide FLPNF-mediated reduction of P70S6K- and S6-phosphorylation was affected by PP2A inhibitor (**Figures 4A–C**).

### Peptide FLPNF Inhibits mTORC1 but not mTORC2 Activity

**Figure 5:**
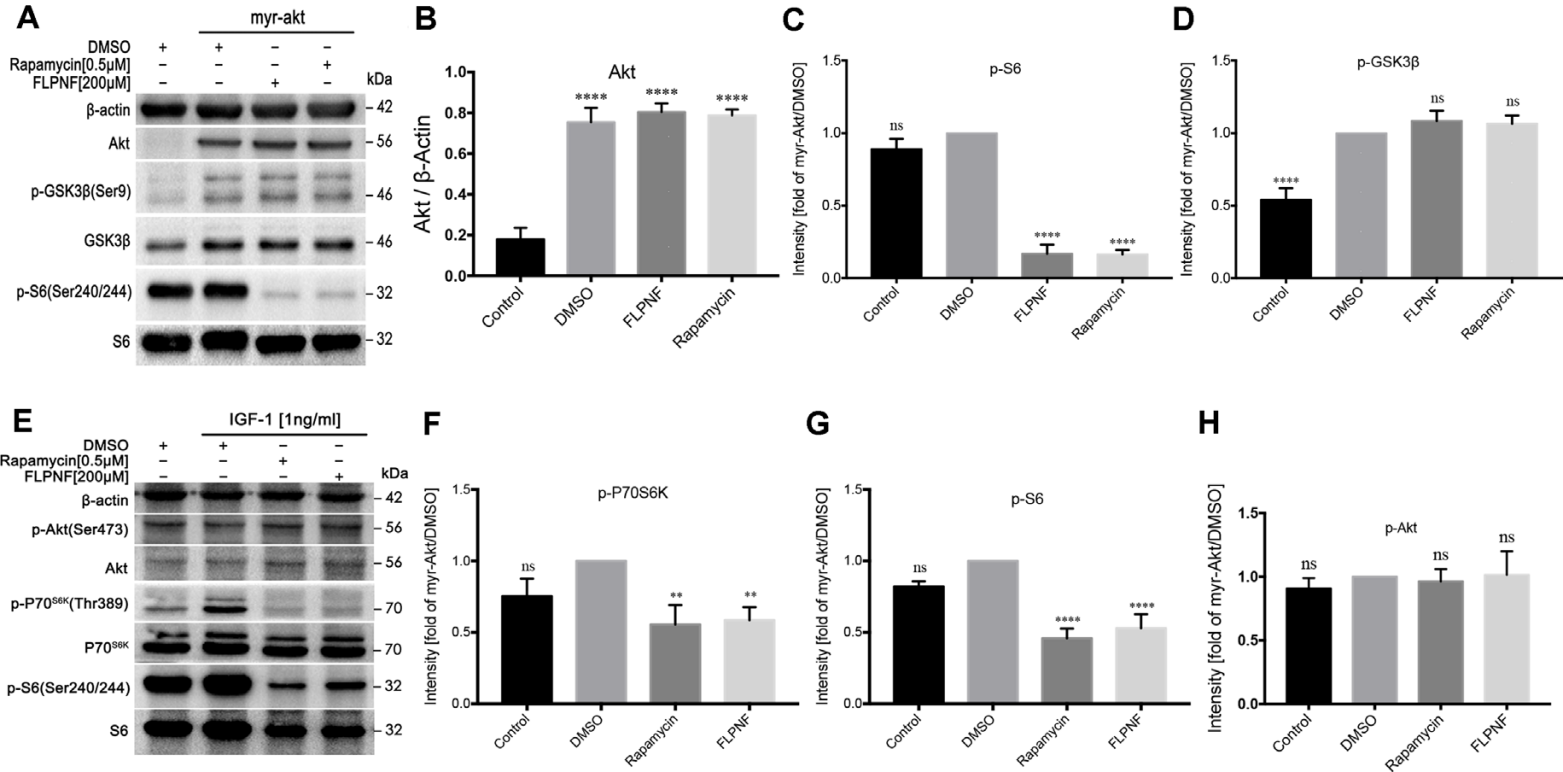
Peptide FLPNF inhibits mTORC1 but not mTORC2 activity. **(A)** Protein levels of Akt, phosphorylated S6, and phosphorylated GSK3β were assessed by Western blot in HEK-293 cells expressing either a control vector or a myr-Akt expression vector after exposure to 200µM peptide FLPNF for 24 h. **(B)** The graph represents the quantification of Akt protein levels (n = 4). ****p < 0.0001 *vs*. control group. **(C)** and **(D)** The graph represents the quantification of phosphorylated S6 and GSK3β protein levels, respectively (n = 4). Band intensities were quantified and statistically analyzed relative to myr-Akt-expressing/DMSO-treated cells. ****p < 0.0001, and ns *vs*. myr-Akt-expressing/DMSO-treated group. **(E)** Protein levels of phosphorylated P70S6K, S6, and Akt were assessed by Western blot in hIAPP-INS-1 cells after exposure to 200 µM peptide FLPNF for 24 h. IGF-1 (1 ng/mL) was added for the final 20 min. **(F**–**H**) The graph represents the quantification of phosphorylated P70S6K, S6, and Akt protein levels, respectively (n = 4). Band intensities were quantified and statistically analyzed relative to IGF-1/DMSO-treated cell. **p < 0.01, ****p < 0.0001, and ns *vs*. IGF-1/DMSO-treated group.

In order to further nail down the specific molecular target of peptide FLPNF in the PI3K-Akt-mTOR-p70S6K pathway, the HEK-293 cells were transduced with lentivirus expressing myristylated Akt (HEK-293-myr-Akt cells). After serum starvation for 48 h, HEK-293-myr-Akt cells increased the level of Akt and P70S6K activity as assessed by the elevated level of phosphorylated GSK3β and S6, respectively (**Figures 5A–D**). The exposure to 200 µM peptide FLPNF for 24 h or 0.5 µM rapamycin for 6 h attenuated the phosphorylation of S6 (**Figures 5A, C**). Conversely, the phosphorylated level of GSK3β, representing Akt activity, was not affected by both compounds (**Figures 5A, D**). Based on the above observations, we concluded that peptide FLPNF, like rapamycin, interferes with PI3K-Akt-mTOR-p70S6K pathway downstream of Akt but upstream of P70S6K.

Since the molecular target of peptide FLPNF resided between Akt and P70S6K within the PI3K-Akt-mTOR-P70S6K signaling pathway, the mTOR kinase activity was assessed. Thus, hIAPP-INS-1 cells were serum-starved for 24 h, then treated with 200 µM peptide FLPNF for 24 h or 0.5 µM rapamycin for 6 h followed by the addition of 1ng/mL IGF-1 in the final 20 min. Both compounds markedly blocked the phosphorylation of P70S6K and S6 (**Figures 5E–G**). In agreement with the previous study, the Ser437-phosphorylation of Akt, as an indicator of mTORC2 activity, was insensitive to mTORC1 inhibitor rapamycin (**Figures 5E, H**). Similarly, peptide FLPNF significantly reduced the activation of P70S6K without disrupting the Akt Ser437-phosphorylation (**Figures 5E, H**). Based on these observations, we concluded that peptide FLPNF increased the autophagy flux *via* inhibiting the mTORC1 but not mTORC2 activity.

### Peptide FLPNF Binds to FRB Domain of mTOR in Molecular Docking Prediction

**Figure 6:**
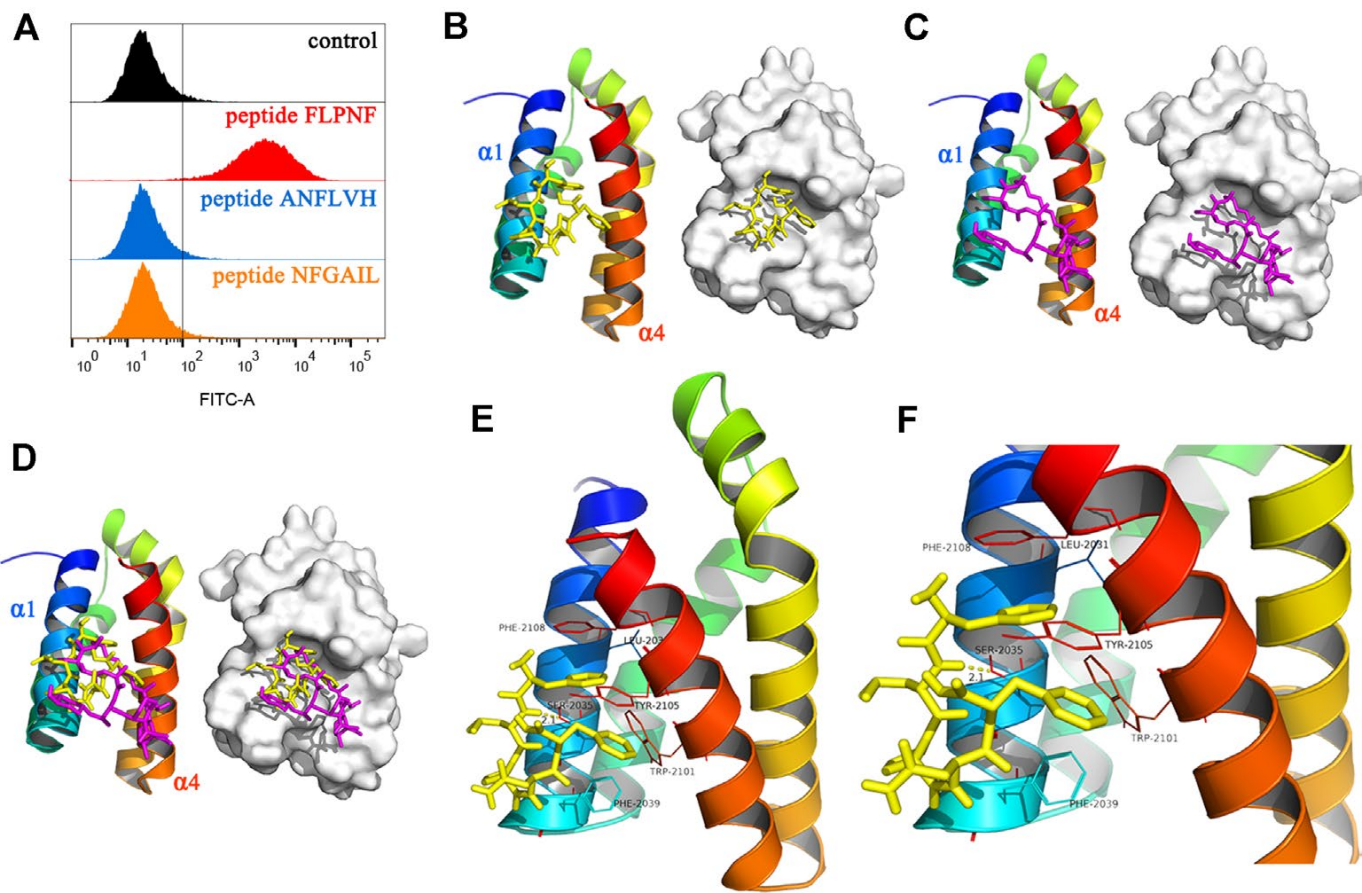
Binding of peptide FLPNF to mTOR and solution structure of the peptide FLPNF-FRB-domain complex. **(A)** Flow cytometry results revealed the peptide FLPNF binds to mTOR. **(B)** The ribbon model (left) and the surface view (right) of the mTOR inhibitor peptide FLPNF docked into the binding site on the FRB domain. **(C)** The localization and conformation of rapamycin bound to the FRB domain in the FKBP12-rapamycin-FRB ternary complex (Choi et al., 1996). In **(D)**, the Peptide FLPNF bound to the FRB domain adopts a position similar to that for rapamycin. The orientation of the FRB domain was same in **(B**–**D)**. **(E, F)** The predicted computational model of the peptide FLPNF-FRB-domain complex. Peptide FLPNF is shown in a stick model, the protein residues are represented in a ribbon model, and hydrogen-bonds appear as yellow dotted lines.

In order to elucidate the mechanism underlying peptide FLPNF-inhibited mTORC1 activity, FITC-labeled peptides were incubated with Flag-mTOR coated magnetic beads, and the fluorescence intensity was measured by flow cytometry. The results indicated peptide FLPNF binds to the mTOR *in vitro* (**Figure 6A**). To further demonstrate the binding site of peptide FLPNF on mTOR, we performed molecular modeling and docking analyses using the Autodock Vina software (Trott and Olson, 2010). The FRB domain of mTOR, which can bind a novel inhibitor of mTOR in the absence of FKBP12, is important in small molecule-mediated regulation of mTOR (Veverka et al., 2008). Therefore, we explored the possibility of peptide FLPNF binding to the FRB domain. The residues from α1 and α4 helices of the FRB domain formed the deep hydrophobic pocket in which rapamycin binds (Choi et al., 1996). And according to docking analysis, peptide FLPNF was docked into the FRB domain (**Figure 6B**). The maximum binding affinity between peptide FLPNF and FRB domain was predicted to be -7.5 kcal/mol. For comparison, the location and conformation of rapamycin on the FRB domain in the FKBP12-rapamycin-FRB ternary complex were illustrated (**Figure 6C**), the peptide FLPNF interacts with the FRB domain applies a dramatically similar position to that seen for rapamycin in the FKBP12-rapamycin-FRB ternary complex (**Figure 6D**). As shown in **Figures 6E, F**, the residues Phe-1 and Phe-5 of peptide FLPNF were located at the hydrophobic sites, surrounded respectively by the residues Phe-2039, Trp-2101, Tyr-2105 of FRB domain, and the residues Leu-2031, Tyr-2105, Phe-2108 of FRB domain, forming stable hydrophobic bindings. The π-π stacking interaction was formed between the side chain of the residue Phe-1 of peptide FLPNF and the residue Tyr-2105 of FRB domain, also between the side chain of the residue Phe-5 of peptide FLPNF and the residue Phe-2108 of FRB domain (**Figures 6E, F**). Furthermore, peptide FLPNF forms hydrogen bonds with the atoms of residue Ser-2035 of the FRB domain (bond length: 2.1 Å) (**Figure 6F**). Thus, the docking assays revealed a direct interaction between the peptide FLPNF and the FRB domain of mTOR.

### Peptide FLPNF Reduces hIAPP Oligomer Accumulation and Protects INS-1 Cells From hIAPP Cytotoxicity

**Figure 7:**
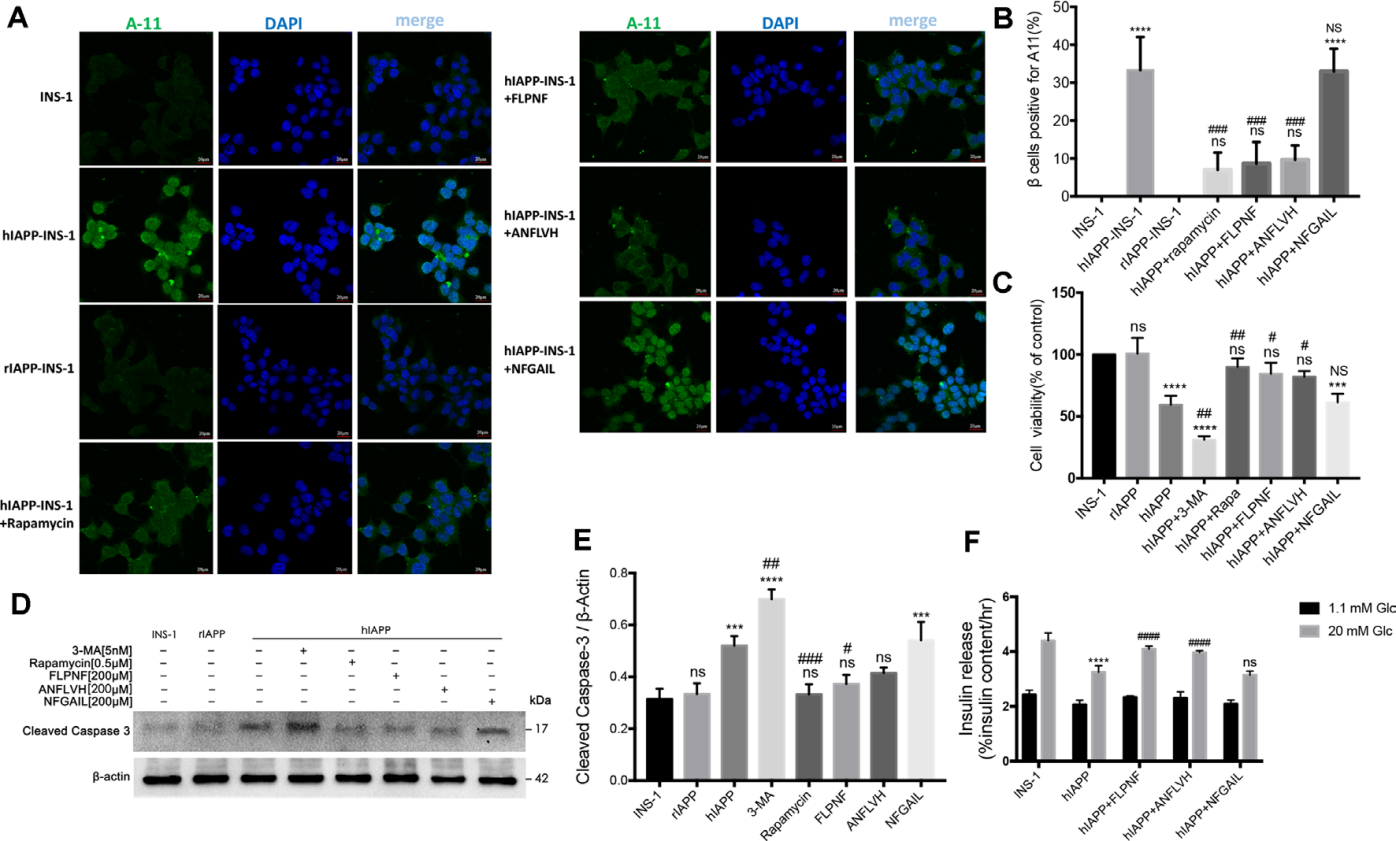
Peptide FLPNF reduces hIAPP oligomer accumulation and protects INS-1 cells from hIAPP cytotoxicity. **(A)** Fluorescence microscopy images of hIAPP oligomer stained with anti-oligomer antibody A11 (oligomer, green; nuclei, blue). **(B)** The quantification of the percentage of β cells positive for cytosolic A11, indicated as bright green puncta deposited around the cell nucleus with larger puncta in the cytoplasm in hIAPP-INS-1 cells (n = 3). ****p < 0.0001 and ns *vs*. INS-1 cells group. ^###^ *p* < 0.001 and NS *vs*. hIAPP-INS-1 cell group. **(C)** The cellular viability of hIAPP-INS-1 cells after exposure to 200 µM peptide FLPNF for 24 h (n = 4). ***p < 0.001, ****p < 0.0001, and ns *vs*. INS-1 cells group. ^#^ *p* < 0.05, ^##^ *p* < 0.01, and NS *vs*. hIAPP-INS-1 cell group. **(D)** The protein levels of cleaved caspase 3 was assessed by Western blot after exposure to 200 µM peptide FLPNF for 24 h, and quantification shown in **(E)**. The band intensities were quantified and statistically analyzed relative to untreated INS-1 cell (n = 4). ***p < 0.001, ****p < 0.0001, and ns *vs*. INS-1 cell group. ^#^ *p* < 0.05, ^##^ *p* < 0.01, and ^###^ *p* < 0.001 *vs*. hIAPP-INS-1 cell group. **(F)** Glucose-stimulated insulin secretion assay of cells after exposure to 200 µM peptide FLPNF for 24 h (n = 4). The data are expressed as percentages of total insulin content. ****p < 0.0001 *vs*. INS-1 cell group. ^####^ *p* < 0.0001, and ns *vs*. hIAPP-INS-1 cell group.

The current data supported the promotion of autophagy flux by peptide FLPNF. Next, we evaluated the hIAPP oligomer and cell viability of hIAPP-INS-1 cells after exposure to peptide FLPNF. Also, the indicator of β cells function, the insulin release, and total insulin content were determined. As shown in **Figures 7A, B**, the toxic hIAPP oligomer, shown as green puncta, was deposited around the cell nucleus with larger puncta in the cytoplasm in hIAPP-INS-1 cells but not in the INS-1 or rIAPP-INS-1 cells (**Figures 7A, B**), which is consistent with previous study (Lin et al., 2007). As expected, peptide FLPNF, rapamycin, and D-ANFLVH significantly reduced the hIAPP oligomer accumulation (**Figures 7A, B**). Furthermore, peptide FLPNF and rapamycin markedly increased the cell viability (**Figure 7C**) and reduced the cleavage of caspase-3 (**Figures 7D, E**). However, autophagy inhibitor 3-MA aggravated the hIAPP-induced cytotoxicity (**Figures 7C–E**). Finally, peptide FLPNF also increased the insulin secretion levels of hIAPP-INS-1 cells in the high glucose condition (20mM), but does not at low condition (**Figure 7F**). High-glucose induced a greater (1.26-fold increase, *p* < 0.0001) insulin secretion in the peptide FLPNF-treated group than the hIAPP-INS-1 group. Peptide D-ANFLVH, which has been reported to improve insulin secretion in hIAPP-transgenic mice, also increased GSIS of hIAPP-INS-1 cells in high glucose (1.22-fold increase, *p* < 0.0001).

## Discussion

In this study, the pentapeptide FLPNF was shown to reduce the hIAPP oligomer accumulation *via* enhancing the autophagy flux by inhibiting mTORC1 activity, thus improving the β cell function and protecting the β cells against hIAPP-induced apoptosis. hIAPP oligomer accumulation has been considered to be a contributing factor for β cell loss and dysfunction in T2D, while the mechanism underlying amyloid formation is still unclear. Furthermore, the reported mechanism of hIAPP-induced cytotoxicity include the hIAPP-induced formation of reactive oxygen species (ROS), breakage of the cell membrane, activation of the inflammasome, and promotion of the apoptosis *via* initiating the cleavage of procaspase 3 to caspase 3 (Raleigh et al., 2017). Therefore, strategies that reduce the hIAPP oligomer deposition exhibit a therapeutic potential.

Recently, autophagy has been considered to play a role in the degradation of IAPP aggregates, and therefore, in reducing the cytotoxic effects of hIAPP. Consistent with the previous study (Morita et al., 2011), our results indicated that the suppression of autophagy flux by 3-MA significantly enhanced the hIAPP-induced β cell apoptosis, whereas enhanced autophagy by rapamycin resulted in reduced apoptosis, which further confirmed the role of autophagy in hIAPP degradation. However, the defects in autophagy aggravated the cytotoxicity of human amylin, and the overexpression of hIAPP by transduction in β cells has been shown to impair autophagy (Rivera et al., 2011; Rivera et al., 2014; Shigihara et al., 2014). The decreased autophagy flux might be attributed to the ability of hIAPP to disrupt lysosomal membranes, which leads to the release of lysosomal enzymes resulting in β cell loss (Press et al., 2018). Hernandez et al. (2018) recently indicated that the overexpression of hIAPP in INS-1E cells resulted in the hyperactivation of mTORC1 and the inhibition of autophagy flux. Moreover, there has been an increasing amount of literature on small molecules that inhibit mTOR activity (Veverka et al., 2008; Morad et al., 2013; Bajer et al., 2014; Kim and Guan, 2015; Fan et al., 2017). Hence, we described the pentapeptide FLPNF as an autophagy activator. The current results showed that peptide FLPNF significantly increased the autophagy flux, thereby promoting the hIAPP oligomer degradation and protecting the β cells from hIAPP cytotoxicity. Further experiments indicated that peptide FLPNF, similar to mTOR inhibitor rapamycin, specifically inhibited the mTORC1 activity without affecting the mTORC2 activity. The flow cytometry results showed the peptide FLPNF bind to mTOR. Intriguingly, the further molecular modeling and docking analyses revealed a direct interaction between the peptide FLPNF and the FRB domain of mTOR. Taken together, our findings provided the possibility of the pentapeptide FLPNF for further development as a potential therapeutic agent for the treatment of T2D.

Our previous study (Shi et al., 2019) found that peptide FLPNF could interact with hIAPP monomer in solution condition *in vitro*, thus inhibiting hIAPP amyloid formation and protecting INS-1 cell from exogenous hIAPP. In the present study, we revealed that FLPNF could interacted with FRB domain of mTOR to induce autophagy flux, which resulted in decrease of hIAPP oligomer in hIAPP-INS-1 cells and protection of INS-1 cells from hIAPP cytotoxicity. Therefore, peptide FLPNF was the inhibitor for both hIAPP oligomerization and mTOR activity. Although FLPNF could inhibit hIAPP amyloid formation in solution condition, the amount of amyloid deposition increased over time (Shi et al., 2019), which indicated that by directly interacting with hIAPP monomer, peptide FLPNF could not inhibit oligomer or amyloid formation completely. In addition, peptide FLPNF could not interact with hIAPP oligomer or the amyloid. Therefore, peptide FLPNF-induced autophgy plays an important role in the clearance of oligomer and amyloid. In conclusion, peptide FLPNF could exert beneficial effects by binding to hIAPP monomer before oligomer formation, while by inducing autophagy through interacting with the FRB of mTOR to increase degradation of oligomer.

Previous studies have shown that physiological mTORC1 activation is required for the maintenance of normal function and development of β cells (Blandino-Rosano et al., 2017), while chronic mTORC1 hyperactivation resulted from nutrient overload causes β cell failure and insulin resistance in T2D (Bartolome et al., 2014; Yuan et al., 2017). In order to suppress the hyperactivation of mTORC1, a well-established mTOR inhibitor rapamycin has been extensively tested in various diabetic animal models. Acute or intermittent exposure to rapamycin improves glucose homeostasis *in vitro* and *in vivo* (Tzatsos and Kandror, 2006; Tremblay et al., 2007), while prolonged treatment has been related to insulin resistance (Deblon et al., 2012; Shum et al., 2016) and reduced of lifespan in diabetic mice (Sataranatarajan et al., 2016).These controversial results might be attributed to the prolonged exposure to rapamycin in addition to mTORC1 inhibition, which also disrupts mTORC2, resulting in β cell toxicity and insulin resistance (Ardestani et al., 2018; Kezic et al., 2018). Therefore, a dual role of mTOR was suggested, and the use of mTOR inhibitors was restricted. The current results revealed that peptide FLPNF enhanced autophagy, which was similar to that of rapamycin, and further studies on efficiency of peptide FLPNF in diabetic animal models are needed. In addition, the concentration of peptide FLPNF (200 µM) was 400-fold that of the concentration of rapamycin (0.5 µM) with the incubation 24 h *vs*. 6 h, respectively, which indicated a lower efficacy of peptide FLPNF as compared to rapamycin.

Although the present study showed that peptide FLPNF effectively enhanced the autophagy *in vitro*, many peptide or protein with promising pharmacological activities fail to perform convincing effects *in vivo*. This feature might be attributed to low stability, short half-life period, or unexpected toxicity. In order to present peptide FLPNF as a candidate for clinical application, peptide modifications (Di, 2015) and further studies are required, which might reveal the potential therapeutic strategies of peptide FLPNF for individuals with T2D.Furthermore, there exist some limitations in our research: (1) we used an *in vitro* model of diabetes by overexpression of hIAPP in INS-1 cells. Although INS-1 cells overexpressed hIAPP could mimic the phathology of T2D, the concentration of hIAPP required for amyloid deposition in INS-1 cells is much higher than that in the blood of diabetic patients. Besides, the microenvironments and factors that affect amyloid formation are much more complicated *in vivo*. Our next goal is to conduct *in vivo* experiments based on the *in vitro* findings. (2) INS-1 and HEK-293 cells used in this study are immortal cells line, which may different from primary cultured islets. Thus the results derived from INS-1 and HEK-293 cells needed further validation in primary cultured islets. (3) The peptide FLPNF needs further modification for better efficacy.

## Conclusions

Taken together, the current findings revealed that pentapeptide FLPNF enhanced autophagy *via* inhibiting mTOR by direct interaction with FRB domain, and thus, promoting the hIAPP degradation and protecting the INS-1 cells from hIAPP cytotoxicity. Therefore, peptide FLPNF may serve a potentially effective therapeutic compounds for T2D.

## Data Availability

The raw data supporting the conclusions of this manuscript will be made available by the authors, without undue reservation, to any qualified researcher.

## Author Contributions

JZ, JL, and XL designed this study and performed the experiments. AJ, CZ, and YS collected the data. JL, ZY, and WL performed the staining. JL and NS performed the statistical analyses and wrote the manuscript. JL, XL, and JZ contributed to data analysis and revised the manuscript.

## Funding

This work was supported by the National Natural Science Foundation of China (NSFC) (Grant no. 31370989).

## Conflict of Interest Statement

The authors declare that the research was conducted in the absence of any commercial or financial relationships that could be construed as a potential conflict of interest.
