# Supplementary material for: Pentapeptide Protects INS-1 Cells From hIAPP-Mediated Apoptosis by Enhancing Autophagy Through mTOR Pathway
Source: Front Pharmacol. 2019 Aug 9;10:896. doi: 10.3389/fphar.2019.00896 (PMC6697068; doi:10.3389/fphar.2019.00896)
Supplement: Supplementary file 1 [file DataSheet_1.docx]

Supplementary Material

## Supplementary Figures

**
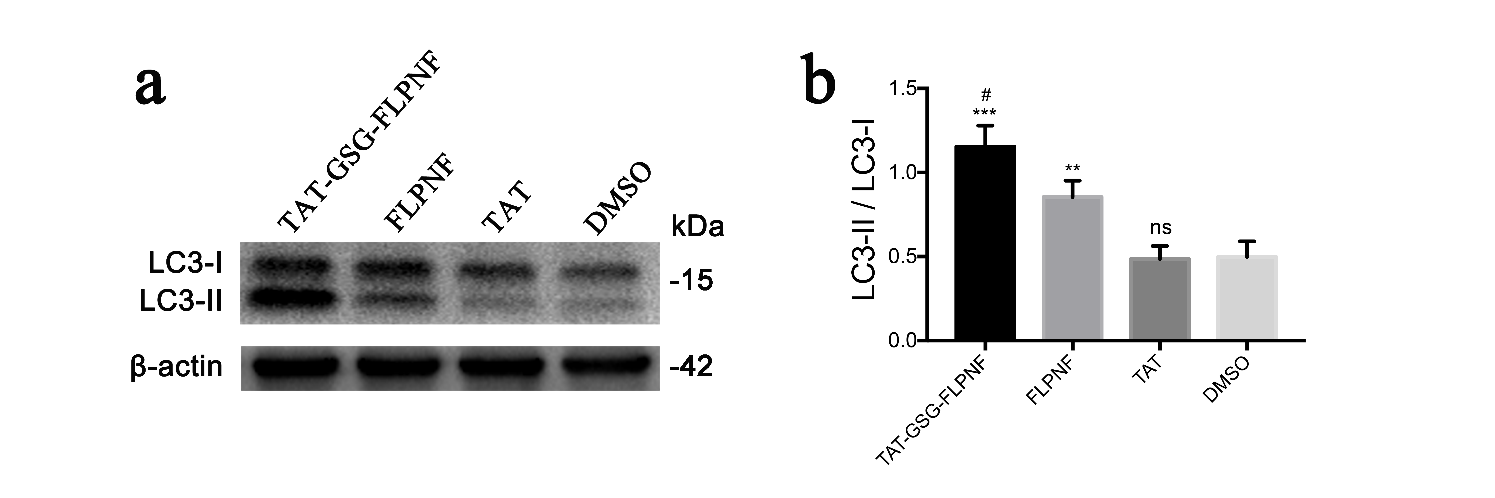
**

**Supplementary Figure 1.** **Both** **peptide FLPNF and TAT-GSG-FLPNF significantly induced the autophagy in INS-1 cells.** INS-1 cells were exposed to 200 µM peptide FLPNF or TAT-GSG-FLPNF for 24 h. Both peptide FLPNF and TAT-GSG-FLPNF significantly induced the autophagy at a concentration of 200μM, and the TAT-GSG-FLPNF was more effective than peptide FLPNF**.** (a) Western blot analysis revealed changes in LC3-I and LC3-II at a concentration of 200 µM of the peptide FLPNF and TAT-GSG-FLPNF. β-actin was used as loading control. (b) The graph represents the quantification of LC3-II protein levels (n=4). **p < 0.01, ***p < 0.001, and ns *vs.* DMSO group. #p<0.05 *vs.* peptide FLPNF group.


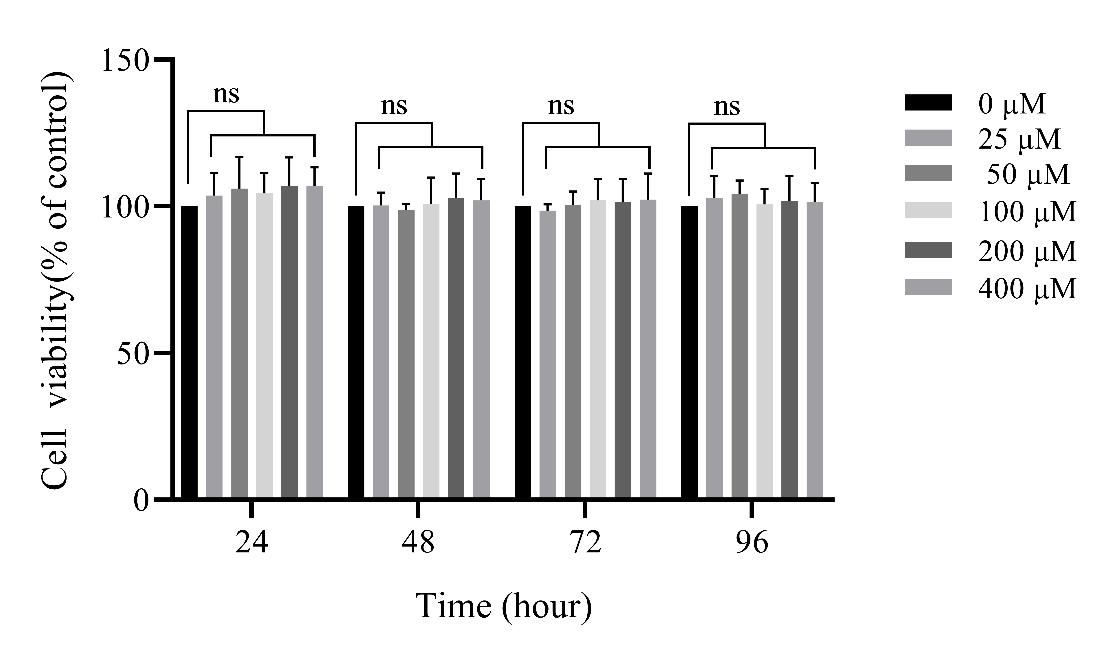


**Supplementary Figure 2.** **Peptide FLPNF hardly affects INS-1 cell viability.** INS-1 cells were exposed to different concentrations (0, 25, 50, 100, 200, and 400 μM) of peptide FLPNF. Cellular viability was determined by CCK-8 assay (n=4). Peptide FLPNF did not cause a decrease in viability of INS-1 cells at different concentrations for different time, even after exposure to 400 µM for 96 hours. ns *vs.* 0 μM group.
